# Child-Centered Design: Developing an Inclusive Letter Writing App

**DOI:** 10.3389/fpsyg.2018.02277

**Published:** 2018-12-06

**Authors:** Marianne Martens, Gretchen Caldwell Rinnert, Christine Andersen

**Affiliations:** ^1^School of Information, Kent State University, Kent, OH, United States; ^2^School of Visual Communication Design, Kent State University, Kent, OH, United States

**Keywords:** co-design, app design, handwriting, human–computer interaction, inclusion, simplified gamification, early literacy

## Abstract

Everywhere there are children, there are screens, and child–computer interaction is ubiquitous. Despite their omnipresence, research on the impact of screens on children’s learning lags behind the development of digital tools. Apple’s App Store has an abundance of “educational” apps, but many of these apps’ claims are unsubstantiated. Organizations responsible for vetting quality products for young people, such as the American Library Association, are developing resources to help identify the best digital products available, but they remain difficult to find, and there is limited guidance for app designers when it comes to designing apps for younger audiences. Our interdisciplinary, empirical study was inspired by “co-creation” (Sanders and Stappers, 2008) and “cooperative inquiry” (Druin, 2005). Starting with a seed grant from Kent State University’s College of Communication and Information, our team sought to create a high-quality and inclusive alphabet app with haptic interactions and simplified gamification to reinforce the basic letter writing skills of young children. The app rewards a child’s successful handwriting with an animation of a verb that corresponds with the letter they traced. Concrete animations and digital and verbal demonstrations connect the typographic letter to the handwritten counterpart. Librarian Claudia Haines’ rubric (Haines, 2016) and the Dig Checklist (Kidmap, 2018.) guided our definition of “quality,” and children served as co-designers in two qualitative user studies. Our young designers tested prototypes, completed task booklets, and were interviewed about their preferences and their feedback informed our design. Additionally, a focus group interview with kindergarten and preschool teachers provided further feedback about the typographic design, stroke order, and gaming rewards. To be inclusive, children in both our app design and user studies were selected from a diverse pool. Our research contributes to work on co-design and cooperative inquiry in the fields of User Experience Design, human-computer interaction, human information behavior, information science, interface design, motion design, typeface design and typography for children, and early literacy development. A post-study is planned upon completion of the app.

## Introduction

The term “Digitods…” according Holloway et al. (2015), “… has been used in education literature to describe those children born after the introduction of the iPhone in 2007” (n.p.). Furthermore, Summers et al. (2013) posit that “(t)hese children often begin their lives with ready access to the Internet via easily usable touchscreen devices, which could have been designed with toddlers’ touch and swipe movements in mind” (n.p.). Most young children today are growing up surrounded by a range of touch devices in the home, yet digital resources for young children face criticism and calls for elimination or restriction from sources such as the Association for American Pediatrics (AAP) and parents. Despite criticisms, the reality is that young children use digital resources in schools as early as preschool, and many parents are using such tools with their children—either because they are fans of technology themselves or because they worry that their child will be behind academically when starting school if they do not have exposure at home (Holloway et al., 2015; Rideout, 2017). While technology is widely used with young children, there are large distinctions between the quality of the apps and how they are used. The authors of this article were frustrated when searching through thousands of apps in Apple’s App Store all claiming to be “educational” in scope. For parents, educators, and librarians alike, finding, evaluating, and selecting quality apps for young children is a challenge, and the App store is difficult to navigate in general. For example, using the terms “ABC” or “Alphabet,” a recent search in the App Store yielded between 279 and 286 alphabet learning apps. To subsequently figure out which app to select or to purchase requires a great deal of effort. Despite the abundance of available apps, there are few resources for evaluating them—and limited guidelines for developing them. Some recently developed resources written by content specialists, such as the American Association of School Librarians’ Best Apps for Teaching and Learning, or the Association of Library Service to Children’s Notable Children’s Digital Media, are intended for practitioners and are not necessarily known to parents. In this study, we used an existing evaluation tool, the KIDMAP framework, as a starting point for creating a quality, educational, learning tool.

In fact, determining what constitutes “quality” within an app is up for debate. It is easier to determine what constitutes a lack of quality—for example, apps that feature in-app advertising, have poor navigation, feature distracting and non-educational gamified elements, or those that infringe upon children’s privacy. To explore what constitutes “quality” within a digital product for young children, we decided to create an app that addresses a fundamental literacy need of preliterate children, between the ages of 3-to-6-years-old—learning to identify and write the alphabet. We had three goals. First, our app would be high quality. We used librarian Claudia Haines’ rubric called Evaluating Apps and New Media for Young Children (Haines, 2016) and KIDMAP’s Dig Checklist (Kidmap, 2018) to help us define “quality” in our app design. Second, we wanted to design an app for young children that addressed their preferences, but was also designed to have an age-appropriate user interface. We integrated children’s opinions by incorporating elements of cooperative inquiry (Druin, 2005) and co-creation (Sanders and Stappers, 2008). Children’s feedback informed our research throughout the design process from how the navigation functioned to how we rewarded their success. Third, we wanted to create an inclusive app for all children. To do so, we ensured that a racially diverse group of children appear within the app and served as co-designers.

Learning to recognize and write letters, which is a foundational building block in developing literacy, is an onerous process. Nicholson (2012) describes meaningful gamification as that which provides internal motivation supporting a user’s learning. While there are many alphabet apps on the market, we felt that experimenting with the genre of abcedaria to work toward creating a high-quality and inclusive digital primer that uses meaningful but simplified gamification for the youngest users—could motivate children to make a repetitive activity interesting and fun. According to Roseberry et al. (2014), many of the alphabet apps on the market lack quality, engagement, and focus almost exclusively on nouns, rather than on verbs. Yet verbs are highly necessary too. In their study on video chat and socially contingent learning, Roseberry et al. (2014) write that “Verbs are the building blocks of grammar and the fulcrum around which a sentence is constructed. Nearly 30 years of research demonstrates that verbs can be significantly more difficult to acquire than nouns for children learning English” (p. 4). And despite the difficulty of acquiring verbs, in their study on assessing vocabulary learning in early childhood, Hoffman et al. (2014), found that a common assessment of children’s vocabulary learning, the Peabody Picture Vocabulary Test (PPVT), actually preferences assessing the acquisition of nouns (72%) over verbs (21%).

We sought to explore verbs for two reasons. First, the touchscreen interface allows for demonstrative animation and incorporates motion design to explain the definition of a word. Second, because existing resources, such as alphabet apps and books tend to focus on nouns, and research, as cited above, points to a lack of focus on verbs, we saw an opportunity for our app to fill a void and tackle a difficult concept for young audiences. By focusing on verbs, our app seeks to expand children’s vocabulary acquisition, especially around their understanding of verbs, as well as their letter writing skills.

Our article begins with a review of the literature that addresses: (1) the ubiquitous nature of technology for young children; (2) conflicting views on the use of technology with this population; (3) the role of parents in selecting and determining use; (4) children’s letter learning in multimodal formats; and (5) app evaluation and guidelines for developers. Next, we discuss our methodology and our user studies, which is followed by our research questions and findings.

### Ubiquitous Nature of Technology for Young Children

“Digital technologies are the new tools—mediating a child’s experience of the world, their language, their physical interactions through cause and effect, and their social interactions. Technology has also become an integral part of many informal learning environments that children encounter more and more often in their lives” (Mills et al., 2015, p. 27).

According to Common Sense Media’s 2017 study, the use of mobile technologies is widespread. Rideout (2017) found that for nearly 98% of children under age 8, there is some type of mobile device in the home. Takeuchi and Stevens (2011, p. 12) point out that “mobile devices and wireless Internet … [allow] media use to be an ‘anywhere, anytime’ phenomenon for families with the financial means to purchase such digital products.” Vittrup et al. (2016, p. 50) found that outside of school or daycare, young children are spending nearly 5 h a day using “some type of media or technology.” In addition to the fact that they are readily available, according to McManis and Gunnewig (2012), mobile touchscreen tablets are designed to be easy and intuitive for even the youngest children to use. Besides, children respond to touchscreens. For instance, Holloway et al. (2015) state that “… a very short time spent in the company of toddlers using touchscreens is sufficient to demonstrate the sheer delight that these young infants have in developing their sense of agency and autonomy.” Yet many researchers point to the fact that the quality of digital media designed for young children is lacking (Fuller et al., 2015). Since we know that “during the first 6 years of life, children experience tremendous cognitive growth in the areas of perception, comprehension, language development, memory, problem solving, and concept representation” (Vittrup et al., 2016, p. 44, citing Siegler and Alibali, 2005), it is imperative that the media we expose them to is of the best quality.

Unfortunately, most of the apps under the “educational” category in the App Store target only rote academic skills, are not based on established curricula, and use little or no input from developmental specialists or educators. According to Hirsch-Pasek et al. (2015, p. 3), “educational” apps found in the App Store are “largely unregulated and untested.” Recently, app evaluation guides have been developed, such as the one by Stone-MacDonald (2014) and another by librarian Haines (2016), whose eleven-question app evaluation rubric for parents, caregivers, and librarians evaluates user experience and content. Haines’ rubric has been further modified by the KIDMAP group to create the Dig Checklist (Kidmap, 2018), which seeks to identify ways that high-quality children’s media is also “inclusive, equitable, and accessible” (Kidmap, 2018), for example, by making sure that such media includes diverse characters, voices, and content.

Experts and parents have conflicting views on the use of digital tools with young children. The American Association of Pediatrics (AAP) has gone from a 2012 recommendation of zero screen time for children under the age of two, to a gradual acceptance with limitations in 2016—in part because parents are using these tools with their young children. As Ferguson (2017) argues, an overly strict stance from the AAP and similar organizations inadvertently fosters the impression of such organizations as being anti-media—if not anti-parent. According to Holloway et al. (2015), parents and caregivers also realize that this is an area that invites criticism and negative judgment by others.

The AAP released a revised policy on screen time in 2016, which softened their original stance and advised pediatricians to counsel parents on developing a Family Media Use Plan that features “unplugged spaces and times in their homes,” using technology in “social and creative ways,” and balancing technology with “sleep, exercise, play, reading aloud, and social interactions” (AAP Council on Communications and Media, 2016, p. 3). The AAP still recommends limiting use for children under the age of two, promotes “Joint Media Engagement,” and warns that there is evidence of harm from excessive use. Other agencies, the U.S. Department of Education (2016) and Health and Human Services, agree with this view.

Despite warnings from authoritative agencies, Ferguson (2017, p. 798) describes screens as becoming more and more integrated into our everyday lives and making “the applicability of such guidelines … less clear.” Instead, it is important to focus on *how* media is used rather than how often. No matter what agencies, such as the AAP recommend, the reality is that “[t]he socio-cultural environment of the current population supports technology use by younger children” (Blake et al., 2011, p. 44).

Recent research has highlighted the possible benefits of digital technologies. Hourcade et al. (2015) analyzed videos of children aged 12–17 months using devices that indicated social use and found that children were just as interested in educational apps as in games and that “…many of the apps appeared to enable children to practice perceptual and motor skills. While tablets could still be used in ways that may lead to negative outcomes, recommendations of no screen time seem exaggerated and based at least in part on incorrect assumptions,” (Hourcade et al., 2015, p. 8). Geist (2012) found evidence that digital technologies could be an aid to educating children, and de Freitas and Prendergast (2015, p. 2) write that “literature about digital media in early childhood, although scant, provides some evidence that some of the unique and very new features of current digital media (most in the form of tablets and developmentally appropriate apps) can and does support early learning in young children.” The National Association for the Education of Young Children [NAEYC] and Fred Rogers Center (2012, p. 7) concur but with the caveat that this cognitive and social learning can occur when technology is used appropriately and when the software is well-designed (de Freitas and Prendergast, 2015, p. 2). It is the position of the Association for Library Service to Children, that when appropriate media is used intentionally, it can promote effective learning (Campbell et al., 2015). Given et al. (2016, p. 347) state that “Using digital technologies requires children to learn and use multi-literacies (a repertoire of flexible skills across a range of media) and in multiple modalities, including written, visual, aural, gestural, linguistic and tactile experience.” Citing conclusions by Takacs et al. (2015), Teepe et al. (2017) found that “multimedia features like animated pictures, music and sound effects were beneficial for children’s productive vocabulary and story comprehension, whereas interactive elements like hotspots, games, and dictionaries were not” (p. 125).

Many parents also believe that the benefits outweigh the risks. Those participating in Rideout’s (2017) study believed that their children benefitted from such media and reported that their children demonstrated improved basic skills, engaged in imaginative play, or had been inspired to engage in other activities. In their 2016 study, Vittrup et al. (2016, p. 52) found that one-third of the parents agreed with the statement that “Media exposure at a young age (0–3 years) is important for early brain development.” Overall, the same parents agreed that children would fall behind in school if their access to tech tools was eliminated in early childhood. Wood et al. (2016) also found that the majority of parents in their study supported using digital media with their children before the age of 2.5 years.

Digital media can also work to support different learning styles. “Technology apps are one tool in supporting children with disabilities’ access to curricular content using the Universal Design for Learning (UDL) framework” (Stone-MacDonald, 2014, p. 5). While the Energetic Alpha app is not currently designed specifically for children with disabilities, modifications could be made in the future to address different learning styles.

Despite recommendations of limiting screen time with young children, it is easy to understand parental anxiety due to the early adoption of touchscreen technology that occurs in formal education. Researchers such as Musti-Rao et al. (2015, p. 165) found that the “adoption of educational apps using touchscreen technology has increasingly become common in today’s K-12 classrooms.” Since children are expected to use digital technologies starting in kindergarten, it is not surprising that parents consider using such technologies in the home helps prepare their children for school.

### Writing as a Process/Letter Learning

According to Schickedanz (1999), children progress through several stages when learning to write the letters of the alphabet. Starting with scribble writing and mock letters, children progress to a few actual letters (usually the first initial of the child’s name), then after practicing more consistently with actual letters, they progress to correct letter writing. During the process, children continually move back and forth between the stages, becoming more comfortable with their relationship to print. In order to move through these stages, children must come to know “(1) a good visual images of each letter…, (2) knowledge of the line segments used to form each letter, (3) knowledge about the sequence in which the lines are put together to compose the letter, and (4) knowledge about the direction in which to draw each of these lines,” (Schickedanz, 1999, p. 109). One specific bit of advice offered by Schickedanz (1999, p. 110) speaks directly to the app: “When children can watch as a letter is written, they gain much more information about the lines used to form that letter.” Further, she points to the need for children to view demonstrations of the correct direction for lines to be made in order to form letters, as this type of knowledge cannot be gleaned simply from viewing letters already formed (p. 111).

Schickedanz discusses the importance of print in all its forms, from environmental print to books to children’s own writing. In a later book, co-authored by Strickland and Schickedanz (2009, p. 77), the authors describe using computers in writing instruction as a way to “extend and reinforce their alphabet knowledge.” This enhancement is possible because computers allow children to meet the task at their own level, in ways that are both engaging and diverse.

The Teacher’s Manual for Phonological Awareness Literacy Screening (PALS) carried out by the majority of schools in Virginia discusses several criteria on which to test preschool children on their pre-literacy skills. Developed for Virginia’s Early Intervention Reading Initiative, PALS allows schools to assess children in order to better design literacy instruction and provide support to those who need it. According to PALS-PreK, a set of skills which are “predictive of future reading success” (PALS-PreK, 2003/2018, para. 1), include name writing, alphabet knowledge, print and word awareness.

Research shows that technology can be beneficial in developing young children’s handwriting skills. “Neumann (2016) found evidence to suggest a positive association between 2- and 4- year olds’ use of touchscreen devices and their print awareness, print knowledge, and sound knowledge, suggesting that these pre-writing activities can promote the development of reading and writing skills” (Kwok et al., 2016, p. 2). Price et al. (2015, p. 2) found that “[t]he use of an iPad also allows 2- to 3-year-old children to produce more continuous and complex mark making (a foundational skill for writing) when compared to the use of traditional paper and paint.” Huber et al. (2016) and Kwok et al. (2016) found that in the case of puzzles, skills learned on touchscreen devices transferred to physical versions of similar tasks (p. 2). Learning to write in multi-modal environments can also be a benefit. According to Mayer (2007), when children learned to write with different materials or on different surfaces, it helped to build fine motor skills and motivated them to write.

### Evaluation of Apps: Points for Developers/Designers of Technology

Research provides many suggestions for those who create and design educational apps for children. In a broad sense, “The best design for children considers their developmental stages when creating classification schemes, hierarchies, metadata, and interfaces” (Martens, 2012, p. 159). In addition, “Well-designed software affords children an appropriate level of control and agency depending on their age and experience, allowing them to proceed at their own pace and sustain interest” (Hirsch-Pasek et al., 2015, p. 10). To accomplish this goal, Levinson et al. (2015) advocate that designers represent diverse backgrounds and languages, and support adults to find and use appropriate media. Takeuchi and Stevens (2011) advocate for joint-media engagement principles that are child-driven and involve multiple planes of engagement as well as co-creation. Taking a slightly different tack, Fuller et al. (2015, p. 18) task developers with advancing three facets of children’s learning and development: “advancing school-related knowledge and cognitive skills, offering tools that spur interaction within families, and linking children and parents to community resources.” They further indicate that developers must consider the culture of the intended audience. Falloon’s (2013, p. 519) study “strongly argues that careful attention should be paid to the design and content of apps if the undisputed motivation from using devices such as iPads is to be transformed into thoughtful engagement and productive learning,” and concluded that designers should endeavor to create apps that include “an appropriate blend of game, practice and learning component.” Miller and Kocurek’s (2017) guiding principles for designing and developing educational games for children under five advocates for the same:

“… games should: (1) [include] developmentally appropriate content; (2) integrate the theoretical frameworks from the learning science field; (3) embed learning in socially rich contexts; (4) develop diverse content; and (5) create a balance between play and real-world learning opportunities. We build the principles around a hypothetical educational game designed to facilitate language development” (Miller and Kocurek, 2017, p. 314).

The U.S. Department of Education (2016, p. 19) offers a useful loop for app developers to follow: create content that is research-based, work with researchers and practitioners to study content and how children respond to it, engage in “a continuous improvement cycle to improve efficacy over time”; and finally, share the findings with others.

### The Role of Libraries

Libraries are key sites for providing access to technology and teaching digital literacy and skills. de Freitas and Prendergast (2015) suggest that libraries create materials to help patrons find quality apps, provide lists and access to them in the library, design programming that introduces patrons to the best apps, and provide an avenue to teach media habits that are healthy for both children and the adults who work with them. Farmer (2004) suggests that librarians incorporate technology in storytelling as a research tool, audio-visual aid, and communication vehicle to improve storytelling. Paciga and Donohue (2017, p. 58–59) state that librarians should “Continue work to curate libraries of exemplars in which educators, child care professionals, and parents can see excellent and developmentally appropriate use of technology and media in contexts that might positively impact a child’s development.”

## Methods: Child-Centered Design

Our child-centered design methodology borrows from Allison Druin’s *co-operative inquiry* (Druin, 1999, 2002) and Elizabeth Sanders’ *co-creation* methodology (2008). Co-operative inquiry is

“…grounded in human-computer interaction (HCI) research and theories of cooperative design [3], participatory design [4], contextual inquiry [5], activity theory [6], and situated action [7]. Cooperative Inquiry is unique from these previous design methods in that it is specifically intended to inform the design process of teams that include adults and children” (Guha et al., 2013, p. 14).

According to Sanders and Stappers (2008, p. 9), “Co-designing threatens the existing power structures by requiring that control be relinquished and given to potential customers, consumers or end-users.” In our design strategy, we sought to relinquish some of the control in order to include the voices of our target audience—children—in the app design. It was our goal, as Guha et al. (2013, p. 18) write, to “…empower children to share their ideas in ways that enable adults to truly hear and appreciate what they are saying.” We also wanted to make sure that our co-designers represented a diverse group of children. “The challenge for researchers is to create a co-design team with children who are as diverse as those in the end-user population” (Martens, 2012, p. 167–168).

While ideally, diversity should encompass children of different ethnic and racial groups, socio-economic levels, abilities, and genders, because of the small size of our study, diversity here is limited to diversity of age, gender, and race. Generally, working with children presents challenges. “(O)ften there is a gap between the contributions from young design partners and the realities of designing a technological artifact” (Frauenberger et al., 2012, p. 2377). We also found that in some cases, the children’s very creative ideas did not correlate with our project’s budget. “Other constraints include how tired the children might be, what days can be planned for design, and how much interaction with the researchers the children are expected to have in the learning environment” (Yip et al., 2013, p. 208). We also experienced scheduling challenges between children, parents, and researchers.

Our process included three studies, of which the first two involved co-design activities with children: a prototype study (User Study 1), a redesigned prototype study (User Study 2), and a focus group with preschool and kindergarten teachers (Teacher Focus Group). All three studies incorporated free play time and an interview period. As Yip et al. (2013, p. 209) wrote, “As a design method, Cooperative Inquiry (CI) includes both observations and interviews as a means of understanding and triangulating children’s design ideas”; we included observation, ethnographic field notes, and interviews in addition to the co-design activities. A task booklet (Appendix C) provided a way to keep interviews on track while providing a space for notes and visual references for the children.

However, we cautiously interpret our findings, as our measures were qualitative in nature and may not translate to other settings.

### User Study 1: Process

This first study involved testing the rough prototype. The process began with testing a brand-new typeface, designed by Aoife Mooney (which we later did not use). In order to design an alphabet, type designers begin with five letters: A, D, H, K, and M, as those letters include all the modular strokes needed to create all upper case letters in the alphabet. In addition, these letters accurately portray most movements a child would need in order to practice the alphabet within our app. At the time of our first study, the interface design was complete but only those first five letters had been programmed to unlock an animation reward. The other 21 letters led back to the main menu. The interface was operating on one iPad mini, one iPad 2 and several 3rd and 4th generation iPads. Our goal was to test the interface, the typeface and then respond to the feedback. By focusing on this limited user study we gained early and definitive feedback, which allowed for quick refinement, without impacting our budget or timeline. This tactic is often used in user experience design, when valuable information can be collected with a limited design prototype and will enable for product development to respond and refine.

**FIGURE 1:**
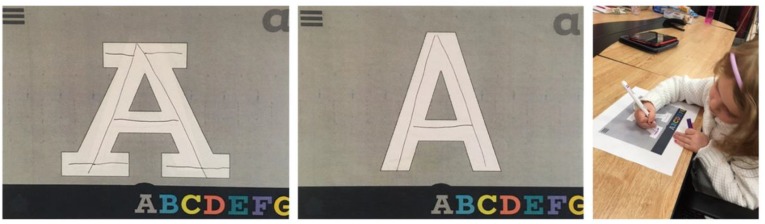
Paper prototype testing with different slab serif and san-serif typefaces.

Our study included the following steps:

(1) Children had free play with the app;
(2) Two researchers conducted one-on-one interviews with children and caregivers;
(3) Children worked on a paper prototyping exercise (see Figure 1);
(4) Co-design activity: children made suggestions for improvements to the app on iPad-shaped sketch paper;
(5) Researchers conducted a follow-up interview with the children to gather their suggestions.

During the session, data was gathered as follows: (1) a doctoral student took ethnographic field notes (Emerson et al., 2011); (2) the photographer took pictures; (3) we asked the children to use the iPad-shaped paper to draw or write what they would like to see in the videos of the app, or to offer their suggestions for improvements to the app overall; and (4) the children interacted with a paper prototype to try different typographic options; and (5) the team interviewed children and their caregivers (Appendixes A, B). Our study included four children ages 4–6 years old and six children ages 7–11, and their caregivers, all of whom had been recruited via snowball sampling. The children in the younger age range represented the target user age for the app. Selecting children in the older range for our study was based on Druin’s (2005) work on cooperative inquiry. Children in this age group are older than those learning to write, which made them both interested in the app design process, and better able to provide constructive feedback than younger users in the target age range.

After free play time, children worked with the team using the paper prototype interface. This relatively inexpensive activity served as a hands-on approach to assess the typography to be used in the app and to test the user interface without updating the development, as shown in Figure 1 below. Initially, we were not sure if we should use a slab serif or san-serif font for the app. It was quickly clear that the younger children struggled to identify slab serif letterforms, and we proceeded with sans-serif letters in our design. The failed prototype gave the team a starting point, and an object to discuss with children and teachers.

**FIGURE 2:**
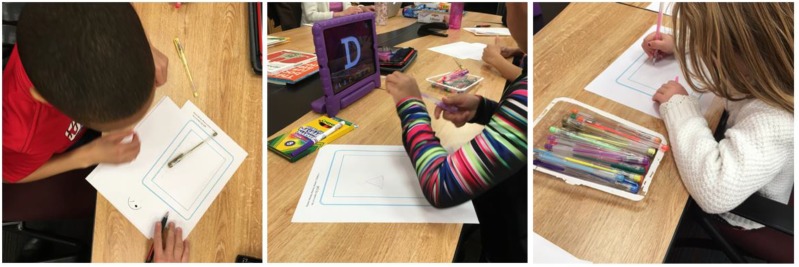
Co-design activity with three participants.

Lastly, children participated in a co-design activity in which they were provided with iPad-shaped paper and asked to sketch the animated reward they would want to see for successfully writing a letter (Figure 2).

Our interview notes, ethnographic field notes, photographs, and observation of the children provided feedback on how they used the interface and responded to the reward videos. Since the sample size of our first user study was very small, we did not calculate inter-rater reliability, and subsequently, our results are limited and not generalizable.

We used Google Sheets (shared only between the researchers) for our analysis, logging results from the interviews, prototype testing, and our observations. The ethnographic fieldnotes added further details and confirmed findings. In addition to verbal responses, children’s notes and sketches on paper prototypes provided suggestions for improvements. In future larger studies, we will include measures of inter-rater agreement. While we used photographs in this study, we did not video record the children, which we would do in a future study.

### User Study 1: Results

There were three findings from the first study:

(1) Our prototype needed to be reconfigured;
(2) The typeface needed to be reconsidered; and
(3) Our users needed error feedback.

While the youngest children were interested in the app, it was a challenge to work with them. For example, one 4-year-old boy repeatedly got up and walked away saying “I’ll be right back, Mommy.” Another was more interested in the many entertaining objects (like puppets, or a cardboard playhouse) in the children’s library center where we did the study; and another would not speak directly to the interviewer, but only to her mother. Overall, shyness was a big factor with younger children, who struggled to provide feedback—especially negative feedback. Compared to the younger group, the older children were more easily focused, easily able to vocalize their criticisms, and were visibly delighted to be included in the process based on their vocal and abundant feedback.

Despite the technical and typographic difficulties, our young users remained engaged in playing with the Energetic Alpha app. Other researchers have found the same in similar studies such as Couse and Chen’s (2010, p. 93) study on stylus-interfaced technology where “… children were seldom frustrated and persisted in their work even when the number of technical incidents increased.” While fully developed, our prototype crashed frequently and we had trouble playing animations during short to moderately long periods of use. All the children found it difficult to write the letters precisely enough to unlock animations, and even when they did, the animations would sometimes fail to play.

The typeface was an immediate concern even before the study began. Initially, we chose a slab serif typeface that was fun and playful, but unfortunately, the more complicated shapes meant that our young and preliterate audience did not recognize some of the letters. The youngest users were not yet ready to compare and understand the relationship between letters with serifs and those without (san serifs). Furthermore, as a tracing guide for handwriting, serif letterforms were confusing. Both younger and older children would fill in the extra serif slabs at the end of the letters. The paper prototyping demonstrated that we needed to change our typeface to a simpler sans-serif font.

Finally, children in both age groups became frustrated when they made errors or had to correct their stroke order. Here is an example from a 6-year-old girl:

When … she couldn’t get the letter to trace correctly, she said, “This is annoying me” over and over and made a surprised face. She slowed down and tried to be precise, but it still didn’t work. “I already did it.” When she finished it, [researcher] asked, “What happened?’ She said, “I don’t know. Now what do I do?” Then she made some funny faces and said, “I’m frustrated” (Field notes, February 17, 2017).

It was evident that we needed to build error feedback into our next design iteration. In addition, neither group of children (younger or older) were sure where to begin writing letters. An 11-year-old girl provided the following feedback: “I don’t think that [younger] kids will understand that they have to follow the red dots – they will think they can do it their own way” (Field notes, February 17, 2017). We realized we needed to insert a visual cue that would signal where to begin making marks.

**FIGURE 3:**
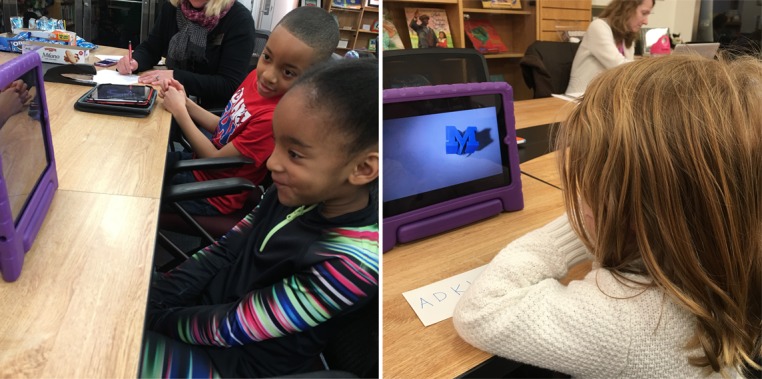
Animation viewing.

We found that the animations held the attention of all users. Even when they did not like an animation, as reported in the final question and answer session, they finished watching until the end of the sequence (Figure 3). When we asked them for input, the older children provided several animation ideas that we were able to incorporate into the final app design. This will be described in the last section.

### User Study 1: Refinement

It took several months to redesign the application. We began by reconfiguring the programming in the app’s backend and developed a new prototype with a simpler iOS programming method. We also chose to host the animations online, which made the app size smaller and within the iOS guidelines. While this decision contradicts Haines’ (2016) guidelines, which suggest that children’s apps should not connect to the Internet, it was necessary for the app to properly function. If all 26 videos had been programmed into the app, it would be too large and would exceed the 4 GB maximum size allowed by the App store. While the writing practice components of the app can be used without the Internet, an Internet connection allows for a more engaging experience. Even when the app is connected to the Internet, child users will not be aware that they are online. There are no in-app purchases or advertising that would take the child out of the app and onto the web.

**FIGURE 4:**
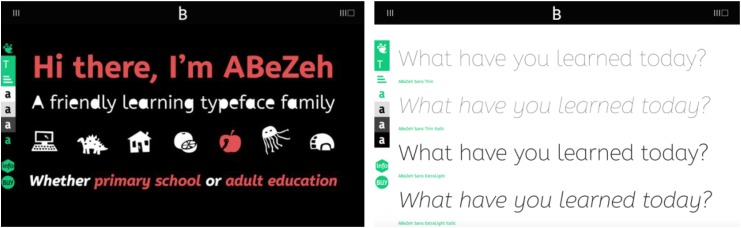
ABeZeh type specimen (bBox, 2018).

Our team selected a new typeface, ABeZeh, designed by Anja Meiners of bBox Type, which is a foundry located in Berlin, Germany (Figure 4). ABeZeh is specifically designed for learning. According to bBox’s website, “ABeZeh’s distinguished, open and friendly shapes simplify the reading (and learning) process – for both adults and children” (bBox, 2018).

**FIGURE 5:**
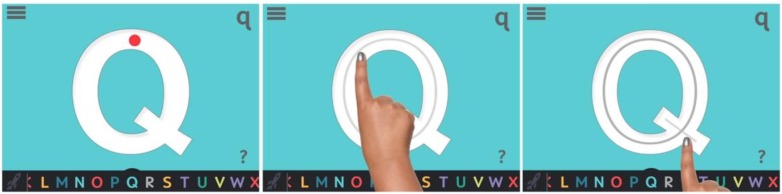
Trace hint animations stills.

To provide error feedback, our team used motion design and sound elements. After User Study 1, we integrated a trace hint animation that played at two moments: (1) when a child attempts to write a letter in the wrong stroke order or (2) when a child waits on the screen and does not begin the action of writing a particular letter (Figure 5). At this point, the trace hint animation plays and shows a child’s hand writing the letter using the proper stroke order while a perfect mark of the letter appears underneath the actor’s hand. The goal is for the child to model the trace hint animation and successfully write the letter.

A variety of motion design animation strategies were used to test which one the children preferred including stop motion, time lapse, vector animations, hand-drawn cell animations, and traditional video recording. We created six additional animations for the letters B, C, F, P, S, and X. Our goal was to use various motion design strategies as a means to appeal to multiple user groups.

### User Study 2: Process

**FIGURE 6:**
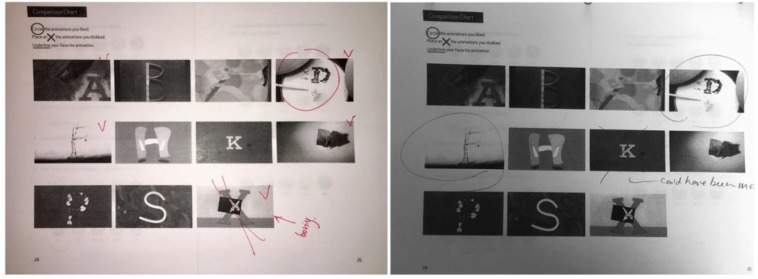
The animation comparison chart from the task booklet with notations from testing.

The second user study included five children aged 4–6 and nine children aged 7–11. The children were interviewed individually or in family groups. There were also three researchers, one research assistant taking notes and a photographer. We used task booklets to guide the interview session (Figure 6). The task booklets organized the study into two separate parts: (1) User Testing and (2) Co-Designing. User Testing covered evaluation of individual letters, error feedback and the basic navigation of the app. The task booklet had three phases:

(1) No sound or animation;
(2) No animation but with sound; and
(3) Sound and animation fully integrated.

Users had an opportunity to define their preferences for interface sounds (success and error feedback sounds). We provided them with an HTML website on which we pushed buttons to play a range of sounds. On the task booklet, we circled their preferences and placed an “X” over sounds they did not like.

We asked the participants about their preferences after they viewed all of the finished animations (11 total) (Figure 6). We provided them with a chart in the task booklet and had them circle the animations they preferred and place an “X” on the animations they did not like. Children had free play with the app, and were able to go back and re-watch any animations they had seen previously. They did not struggle giving feedback—in fact, they were eager to talk about aspects of certain animations. The animation rewards included a wide array of motion design strategies. Each letterform employed a different design method and each was unique. We incorporated hand-drawn illustrations, vector graphics, stop motion, time lapse, photo manipulation, and traditional video recording. Overall, children expressed preferences for longer videos, and they especially enjoyed stop-motion animation.

**FIGURE 7:**
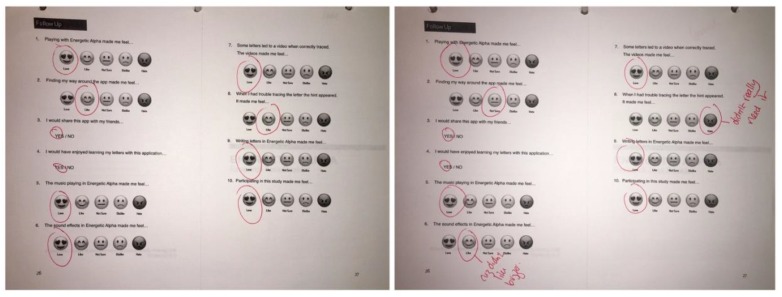
Final page of the task booklet Apple Color Emoji (2017).

We finished the study by asking the children whether they enjoyed the app and if they would recommend it to a friend. The questions had answers that were based on a child-friendly Likert scale and allowed for a range of answers to be shared (Figure 7).

### User Study 2: Results

We found that children required concrete feedback scenarios that utilized sound to accurately self-correct. When we conducted the test without sound, the children were not as quick to understand the interface or to figure out how to self-correct stroke order errors. Sound as an element is always an important feature in interface design, but we found it especially helpful for our preliterate learners. A buzzer provided feedback that was irritating to some children but immediately told the user they needed to self-correct in the same way an adult working one-on-one with a child practicing letter-writing might correct them with verbal commands and gestures.

We also discovered that concrete feedback in the form of visual cues played an important role. Upon testing the new prototype, we realized that the addition of sound and trace hint animations made it easier for children to understand the required tasks, to determine how to navigate the interface, and provided a much better user experience than one without these elements.

In addition to being entertaining, animation can also provide motivation and engagement for young learners that can extend the learning experience beyond skill building and into the reward-experience. We were not surprised to find that pairing animations with letterforms provided strong motivation to continue using the app, and essentially, practice letter writing. Users were still engaged with the animation rewards and continued wanting to discover more even after they had watched all of the completed animations. It was also interesting that children even watched animations they claimed not to like from beginning to end.

Some children were interested in the stop-motion, whereas others were drawn to the vector-style animations. Since the design strategy was intended to be eclectic and allow children to sample new formats, it also permitted surprise and anticipation. While there was not a definitive answer for which animation style they preferred, we did find that children preferred longer animations with more narrative elements. The shorter animations, such as those for letters “K” and “X,” averaging about 10 s each, were the animations that children complained about. In general, children preferred longer animations that averaged 30 s to 2 min. Shorter animations (8–10 s) were considered “boring” or “disappointing.”

Finally, there was much agreement among the children regarding sounds. To signal errors, we were using a loud, game-show-style buzzer. However, after hearing the children’s feedback, we quickly ruled this sound out; children found it to be an upsetting and distracting noise. Instead, they preferred a lower tone that had an electronic beat.

### Teacher Study Process

We conducted a small focus group study of the app with two pre-kindergarten teachers and one kindergarten teacher at the University’s Child Development Center. Two researchers met the teachers in a conference room at the end of the day and after the children had left. Like the children, the teachers were able to experience free play with the app and explore the interface and animations. As the children had experienced in the first user study, the app crashed repeatedly and the teachers experienced a few stroke order errors. In many ways, this served as a short beta testing session. The teacher study confirmed the results from our first two studies. Like the children (described in the next section), teachers also saw a need for competition or success tracking. They suggested adding a feature that would let the child practice writing their name, and we hope to do this in the next version of the app. One teacher suggested a visual effect, like stars, to show the children how well they are doing. Teachers also critiqued the content choices of the animations. They felt that one of the first animations created for the app, “A is for abstract art,” was too complex for preschoolers and pointed out that art is a difficult word as preschoolers do not hear the “a” sound in “art” and only hear the “rt” sound. One teacher informed us that since handwriting is no longer part of school testing in the United States, many schools are choosing to make handwriting a low priority in the overall curriculum.

### Updating Energetic Alpha

**FIGURE 8:**
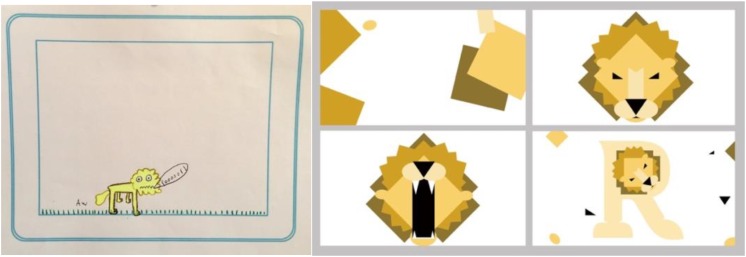
A child’s animation during User Study 1; an image of a lion roaring **(left)** and stills from the final animation for the letter R titled “R is for Roar” **(right)**.

**FIGURE 9:**
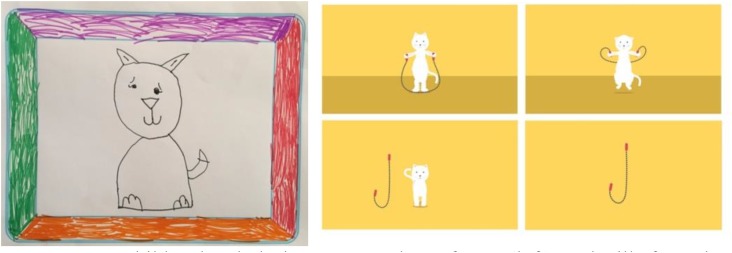
A child’s sketch during User Study 1 of a cat **(left)**, and stills from the final animation titled “J is for Jumping” **(right)**.

We were able to utilize many of the children’s suggestions and feedback. The most interesting changes and suggestions came from our User Study 1, when we gathered children’s ideas for animations. Their feedback was very helpful in designing the remaining animations. For instance, one boy suggested we include a lion, and as a result, the animation for R became “R is for Roar” (Figure 8). Another child suggested we include a cat. When we designed the animation for the letter J, we again used this child’s suggestion and designed “J is for Jumping” using a jumping cat as the character instead of a person (Figure 9).

**FIGURE 10:**
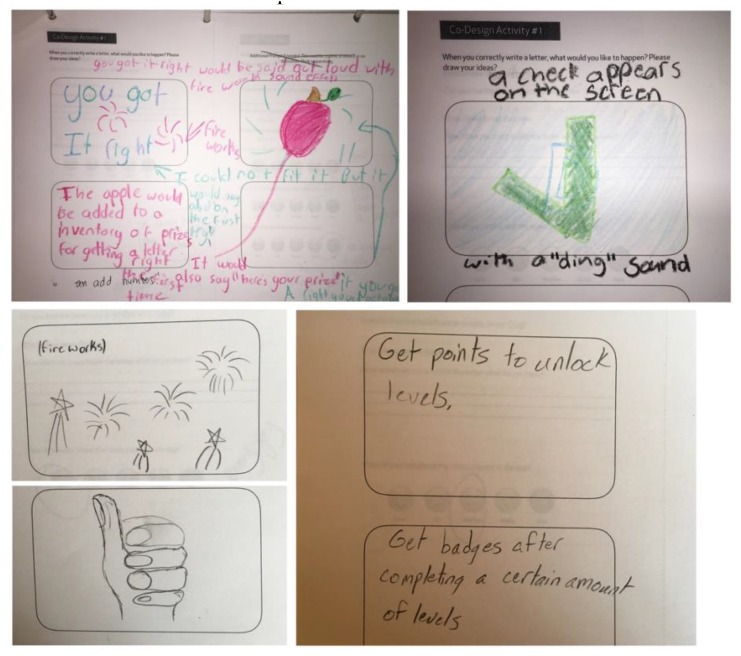
Various sketches from children during User Study 1 showing fireworks and “thumbs up” as well as checkmarks and messages requesting “points” or badges.

We also found that the children wanted some competitive gameplay elements that would provide additional affirmation of success. One child suggested we use fireworks upon successful completion of a task, whereas another proposed a growing pile of apples to represent accomplishments. One child suggested adding a visual check mark along with a sound that would signify success and another suggested a “thumbs up.” An older child wanted to see a leader-board and gamified levels within the game, and suggested that different levels should unlock badges (Figure 10). While these suggestions are appropriate for an older child used to playing video games, based on this feedback, we did incorporate a simple gamified element, as described below, in which a child competes against him or herself, while showing progress to both child user and adult supervisor.

**FIGURE 11:**
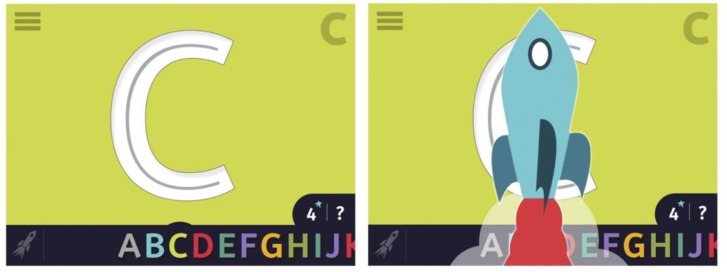
The new interface design with a new counter feature in the bottom right corner of the interface **(left)** and a rocket animation that states “good job” during blast off **(right)**. Children can press the question mark in the lower right-hand corner for “help,” which will then launch the trace hint animation (see Figure 5). Alternately, if there is a delay in game play, help appears automatically.

A success tracker in the right corner of the interface shows how many times a child has completed a letter. We also created incremental rewards for completing one stroke. After a child writes a letter, a star-burst appears along with a “ding” sound. When they complete a letter, they are rewarded with a video. If they are offline or have rewards turned off, then they get the “good job” rocket (Figure 11).

Finally, we integrated the results from the teacher study by addressing all of the technical issues and creating competitive play features as already described above, and the animations for A and S were redesigned according to the children’s suggestions.

### Limitations and Future Research and Development

Our findings were limited by having a small number of users. Even though we created an inclusive app and included diverse children and adults in the app design and in our user studies, the researchers were all Caucasian. In future studies, researchers should also include a diverse group of scholars.

It would also be useful to test children (especially those in the youngest group) using the app over longer time. This would allow us to see if, as children become familiar with the app and proficient in using it, they prefer shorter or longer videos, or at which point children become bored with the animations and seek variety. In addition, a future study might examine whether any letter writing fluency gained via using the app, translates to handwriting fluency in print.

Writing proficiency is an important skill that begins with understanding how to read and write letters and form letters quickly. According to Graham (2009):

“If children cannot form letters—or cannot form them with reasonable legibility and speed – they cannot translate the language in their mind into the written text. Struggling with handwriting can lead to a self-fulfilling prophecy in which students avoid writing, and see themselves as not being able to able to write, and fall further behind their peers” (p. 20).

Another limitation was having to rely on an Internet connection for the best user experience. While an initial goal of our project was to create an app that worked without an Internet connection, we found that programming 26 videos into the app exceeded the maximum size allowed by the App Store. While the app does work without Internet access, rewarding children with a rocket, rather than a video for correctly completing a letter, the gamified elements certainly work best when online.

## Conclusion

Our goal with this project was to create a digital letter writing tool for young children. Since we know that young children are interested in handheld devices, such as iPads and mobile screens, we sought to create a high-quality tool that combined simplified gamification with technology in order to make a monotonous task—letter writing practice—entertaining. Despite the fact that it is no longer emphasized in school, our teacher study showed that at least these teachers still believe that handwriting is important. We set three goals for our study: (1) to make a high-quality app; (2) to create an age-appropriate interface; and (3) to create an inclusive app for all children. By using Haines’ (2016) rubric and the Dig Checklist (Kidmap, 2018) to assess quality, and borrowing elements from Druin’s (2005) cooperative inquiry, and Sanders and Stappers’ (2008) co-creation, our child-centered design methodology guided our study, and allowed us to create an inclusive tool with a diverse group of children. Working directly with participants allowed us to design specifically for them and with them. It also gave us an understanding of the iterative process needed for the design of the children’s app and of design research that utilizes co-design. Even though it was time-consuming and required the team to revise and refine the app, we feel that the end result is a better, more child-centered product. Energetic Alpha was released in the App Store in Spring 2018. Unlike publishing a book, the launch of an app is anything but “final.” Based on reviews and user feedback, we anticipate updating the software soon after release to improve the app’s performance and to integrate ideas that expand usability for new users and address different learning styles.

## Ethics Statement

This study was approved by Kent State University’s Institutional Review Board. The teachers and parents signed a consent form after reading an informational letter and discussing the research with the research team. The parents signed a consent form allowing their children to participate. This form included an audio, video and photography consent, along with a privacy and confidentiality form. The original form stated: “Identifiable information will not be made available in the publications and/or presentations of the research date. Due to this, we obtained additional photography permissions from children who are identifiable in this article. Parents were asked to provide written approval of having their child’s photographic image published in conjunction with this research. Parents of both children who are identifiable, approved and signed additional permission forms.”

Doug Delahanty, IRB Chair, Kent State University

Institutional Review Board

Tricia Sloan, Administrator, Kent State University

Institutional Review Board

Kevin McCreary, Assistant Director, Kent State University

Institutional Review Board

Paulette Washko, Director, Kent State University

Institutional Review Board

## Author Contributions

MM is the lead author, led the initial research design, collaborated on co-design activities, and co-wrote and edited the article. GR is the app creator, designer and art director for the Energetic Alpha iPad App, She led co-design activities, and co-authored and edited the article. CA drafted the literature review, took extensive field notes during testing, and helped with copy-editing and proofreading.

## Conflict of Interest Statement

The authors declare that the research was conducted in the absence of any commercial or financial relationships that could be construed as a potential conflict of interest.
