# Supplementary material for: Child-Centered Design: Developing an Inclusive Letter Writing App
Source: Front Psychol. 2018 Dec 6;9:2277. doi: 10.3389/fpsyg.2018.02277 (PMC6291515; doi:10.3389/fpsyg.2018.02277)
Supplement: Supplementary file 1 [file Data_Sheet_1.docx]

Appendix A

Interview Protocol (Children)

## Interview Protocols for Children

## Evaluation of Animated Alphabet App^[[1]](#footnote-1)^

*Please note that these questions are primarily intended as prompts, or as general discussion guidelines. The conversation may go in slightly different directions in each case.*

**Part I: Basic Demographics**

1. How old are you?
2. What grade are you in?
3. What kind of school do you go to: public / private / or are you homeschooled?
4. Gender identification: male or female?
5. What language(s) do you speak?
6. Who brought you here today?

**Part II: Technology Use:** iPad / Android Tablet Experience

| Have you ever used an iPad or an Android tablet? (If not iPad, what kind do you use)? |  |
| --- | --- |
| Where do you use iPads or Android tablets? (Home, school, or other?) |  |
| What sorts of things do you (or would you) use an iPad or Android tablet for? |  |
| What is your favorite thing to do on an iPad or Android tablet?? |  |
| What sorts of things do you think are frustrating about using iPads or Android tablets? |  |

**Learning to write:**

Can you tell me about how you learned to write letters? Who taught you? How old where you?

___________________________________________________________

___________________________________________________________

**Part III: Interactive Portion**

1. Tell me what you see on this first screen: _________________________

___________________________________________________________

1. Please select the Cyrillic letter provided. What do you think you should do next?

*For Researchers:*

| *Questions:* | *Yes* | *No* |
| --- | --- | --- |
| Did child successfully select the Cyrillic letter? |  |  |
| Did child trace the letter in the correct order? |  |  |
| Did the child struggle to understand the directions? |  |  |
| Did the child follow the trace hints provided? |  |  |
| Did the child successfully trace the letter? |  |  |
| Was the parent engaged in the process? |  |  |

1. Please select the capital letter “D.” What do you think you should do next?

*For Researchers:*

| *Questions*: | *Yes* | *No* |
| --- | --- | --- |
| Did child successfully select the capital D? |  |  |
| Did child trace the letter in the correct order? |  |  |
| Did the child struggle to understand the directions? |  |  |
| Did the child follow the trace hints provided? |  |  |
| Did the child successfully trace the letter? |  |  |
| Was the parent engaged in the process? |  |  |

1. Watch the animation.

*For Researchers:*

| *Questions:* | *Yes* | *No* |
| --- | --- | --- |
| Did the animation play? |  |  |
| Did the animation title appear? |  |  |
| Was the child engaged/interested? Did it hold the child’s attention? |  |  |

1. Can you return to the main screen? Was the child successful?
2. Please select the lowercase letter “a”.

*For Researchers:*

| *Questions:* | *Yes* | *No* |
| --- | --- | --- |
| Did child successfully select the letter “a”? |  |  |
| Did child trace the letter in the correct order? |  |  |
| Did the child struggle to understand the directions? |  |  |
| Did the child follow the trace hints provided? |  |  |
| Did the child successfully trace the letter? |  |  |
| Was the parent engaged in the process? |  |  |

**Part III. Follow-up Questions**

Please use the table to indicate your agreement with the following statements:

|  | Strongly Agree | Agree | Neutral | Disagree | Strongly Disagree |
| --- | --- | --- | --- | --- | --- |
| I enjoyed using Energetic Alpha. |  |  |  |  |  |
| I learned how to draw the new letter using this app. |  |  |  |  |  |
| It is easy to find your way around the app. |  |  |  |  |  |
| I had trouble understanding the animation hints. |  |  |  |  |  |
| I would want to share this app with my friends and family. |  |  |  |  |  |
| I would prefer to use this app on my own. |  |  |  |  |  |
| I enjoyed the animation that played when I unlocked the letter “D”. |  |  |  |  |  |
| I enjoyed the animation that played when I unlocked the letter “a”. |  |  |  |  |  |
| I would have enjoyed learning my letters using this app. |  |  |  |  |  |

1. Is there anything you would change about the app?
2. If there is, can you draw a picture of what you would change?

*(Researchers will provide crayons, paper, markers, pencils, etc.)*

1. Please provide any other ideas or comments you want to share.

Appendix B

Interview Protocol (Caregivers)

## Interview Protocols for Caregivers

Evaluation of Energetic Alpha^^[[2]](#footnote-2)^^

*Please note that these questions are primarily intended as general discussion guidelines. The conversation may go in slightly different directions in each case.*

**Part I: Basic Demographics**

1. What is your age? Please check the box that best applies:

| 25 or under |  |
| --- | --- |
| 30 or under |  |
| 35 or under |  |
| 40 or under |  |
| 45 or under |  |
| 50 or under |  |
| over 50 |  |

1. Gender Identification? __________________
2. What is the primary language spoken in your home? ____________________
3. Are other languages spoken in your home? If so, which ones? ____________
4. What is the highest level of education you have completed? Please check the box that best applies:

| Grammar School |  |
| --- | --- |
| High School or equivalent |  |
| Vocational/technical school (2 years) |  |
| Some college |  |
| Bachelor’s |  |
| Master’s |  |
| Ph.D. |  |
| Professional degree (MD, JD, etc). |  |

1. How many children do you have?
2. How old are your children?___________
3. What is your relationship to the child you brought today?

**Part II: Technology Use**

1. Please put an “x” in the box that best describes your agreement with the following statement: “I am skilled at using technology.”

| Stongly Agree | Agree | Neutral | Disagree | Strongly Disagree |
| --- | --- | --- | --- | --- |
|  |  |  |  |  |

1. Please put an “x” in the box that best describes how you would complete the following sentence: “I primarily use the Internet for ____________”

| Work |  |
| --- | --- |
| Education |  |
| Information Gathering, Research |  |
| Entertainment |  |
| News |  |
| Shopping |  |
| Communicating with Others/Social Media |  |
| Other (please write in) |  |

1. Please put an “x” in the box that best describes how long you have used the following tech tools:

| Length of time using: | Computers | The Internet | Tablets | Other Devices (such as smart phones) |
| --- | --- | --- | --- | --- |
| Less than 6 months |  |  |  |  |
| 6-12 months |  |  |  |  |
| 1-3 years |  |  |  |  |
| 4-6 years |  |  |  |  |
| 7-10 years |  |  |  |  |
| 11-15 years |  |  |  |  |
| 15 years + |  |  |  |  |

4. What types of devices do you have in your home – please check all that apply:

| Desktop computer |  |
| --- | --- |
| Laptop |  |
| iPad |  |
| Android tablet |  |
| iPhone |  |
| Android Smart Phone |  |
| Other |  |

1. Which of these devices do you use with your child(ren)? Could you tell us about how you use them with the child(ren), or if you do not use them with children, could you tell us about your decision?

| computer |  |
| --- | --- |
| tablet |  |
| smart phone |  |

**Part III: Follow-up Questions:**

1. How might you use Energetic Alpha app with your child(ren)? Will they want to play it multiple times?

2. Did you find the app to work as expected? Why or why not? Did the screens and navigation make sense? Was anything confusing?

3. Did your child(ren) appear frustrated with the app at any time? Why do you think they were frustrated? Could they navigate around the app easily?

4. Did you like the design of the app? Why or why not? Was it a streamlined design or was it cluttered?

Please check boxes below to indicate your agreement (or disagreement) with the following statements:

|  | Stongly Agree | Agree | Neutral | Disagree | Strongly disagree |
| --- | --- | --- | --- | --- | --- |
| I believe Energetic Alpha was able to teach my child how to write a new letterform. |  |  |  |  |  |
| I enjoyed using Energetic Alpha. |  |  |  |  |  |
| I learned how to draw the new letter using this app. |  |  |  |  |  |
| It is easy to find your way around the app. |  |  |  |  |  |
| I would want to share this app with my friends/family |  |  |  |  |  |
| I would use this app by myself [directed at the child] |  |  |  |  |  |
| I enjoyed the animation that played once I unlocked the letter “D.” |  |  |  |  |  |
| I enjoyed the animation that played once I unlocked the letter “A.” |  |  |  |  |  |

**Other:**

Is there anything you would change about the app?

If there is, can you draw a picture of what you would change?

- - 1. (provide crayons, paper, markers, pencils, etc.)

Do you have any ideas or comments that you want to share?

Appendix C

Task Booklet

The complete task booklet is available here: <https://drive.google.com/file/d/102bXvDhFT-Gmtv7irrbKTsbGBplJz2Y0/view?usp=sharing>

1. Interview questions are modified from adapted from Massey et al. (2005), Bilal and Bachir (2007), Haines (2016). [↑](#footnote-ref-1)
2. Interview questions are modified from Flowers, S. (2012). Evaluating teen services and programs. Chicago, IL: United States: Neal-Schuman; Haines (2016). Evaluating Apps and New Media for Young Children: A Rubric. [↑](#footnote-ref-2)
